# Cellulose/Aminated Multi-Walled Carbon Nanotube Nanocomposite Aerogels for Oil Adsorption

**DOI:** 10.3390/polym17070869

**Published:** 2025-03-24

**Authors:** Runlin Han, Zihan Liu, Faxiang Feng, Shi Su, Guilin Dong, Xiaobing Liu, Hongbo Gu

**Affiliations:** 1 Key Laboratory of Jiangxi Province for Special Optoelectronic Artificial Crystal Materials, School of Chemistry and Chemical Engineering, Jinggangshan University, Ji’an 343009, China; hanrunlin@163.com (R.H.); fengfaxiangfj@163.com (F.F.); 178885271280@163.com (S.S.); dong2990455445@163.com (G.D.); liuxiaobing805@163.com (X.L.); 2 Key Laboratory of Chemical Assessment and Sustainability, School of Chemical Science and Engineering, Tongji University, Shanghai 200092, China; 15638112382@139.com

**Keywords:** aerogel, cellulose, aminated multi-walled carbon nanotubes (MWCNTs-NH_2_), composites, oil adsorption

## Abstract

At present, the oil extraction and chemical industry and other industries produce a large amount of oily wastewater and organic sewage, and the world is suffering from oil spills and organic wastewater pollution. As a porous material, aerogels are promising in the field of oil adsorption. In this work, the nanocellulose/aminated multi-walled carbon nanotube (NC-MWCNT-NH_2_) nanocomposite aerogel with a high porosity of up to 97.80% is prepared by varying the weight percentage of MWCNTs-NH_2_, among which the nanocomposite aerogel with 0.1% weight percentage of MWCNTs-NH_2_ exhibits the best adsorption performance with the adsorption capacity to cyclohexane, ethyl acetate, anhydrous ethanol, methylene dichloride, acetone, kerosene, pump oil, and used pump oil of 39.77 ± 0.82, 44.54 ± 1.67, 43.03 ± 1.06, 62.13 ± 0.36, 39.92 ± 1.09, 39.37 ± 0.27, 43.48 ± 0.06, and 38.45 ± 0.84 g·g^−1^, respectively. Compared with pure nanocellulose aerogel, the adsorption capacity of the NC-MWCNT-NH_2_ aerogel to pump oil is improved by up to 93.9%, which exhibits excellent adsorption properties and could be utilized in the field of oil adsorption.

## 1. Introduction

The rapid development of industries such as petrochemicals and production has promoted the rapid growth of the country’s economy, but it has also brought a lot of environmental problems [1,2,3]. Oily wastewater refers to wastewater discharged from industrial production containing oil, like petroleum-related products, tar and its fractions, fats, and edible animal and vegetable oils [4]. There is an urgent need to develop innovative technologies and materials that are sustainable and scalable for the efficient collection and separation of spilled oils and organic solvents from contaminated water sources. Chemical methods such as flocculation and precipitation are often expensive and complicated. Also, these methods are not environmentally friendly [5]. The adsorption method uses solid porous adsorbents such as activated carbon, polypropylene fibers, and polyurethane foams that are commonly used to clean up oil spills. It is one of the most promising methods for its high efficiency, simple operation, relatively lower cost, less secondary pollution, and higher recyclability [1,6].

Aerogel is a nanoscale porous solid material formed by replacing the liquid phase of a gel with gas, and is the lowest-density solid material known to exist on earth [7,8]. In addition to this, it has a compressive capacity equivalent to thousands of times its own weight, extremely high thermal and insulating properties, and a low refractive index. Aerogels have extremely high porosity, a high specific surface area, and an ultra-high pore volume ratio [9].

Cellulose is one of the world’s most abundant and renewable resources. It is a linear homopolysaccharide composed of *β*-D-glucopyranose units linked by *β*-1-4 linkages, with highly ordered crystalline phases as well as amorphous domains such as lignin, pectin, hemicellulose, and so on [10]. Cellulose aerogels combine the advantages of aerogels with low density, high porosity, large specific surface area, and excellent biodegradability [11]. It also has good adsorption properties, which could adsorb oil substances and greatly benefit energy saving and carbon emission reduction [12]. Cellulose nanofibrils (CNFs) are composed of a long, flexible, and interconnected network of cellulose nanofibers, with a diameter of 2–60 nm and a length of several micrometers, containing both crystalline and amorphous cellulose domains. CNF aerogels have a broad range of functional applications, such as efficient adsorption, thermal insulation, and energy storage [13,14,15]. Zhang et al. prepared a highly compressible and hydrophobic aerogel by freeze-drying of the aqueous suspensions of CNFs, which showed a hexane absorption capacity of 45 g·g^−1^ [16]. Zhang et al. [17] used nanocellulose as a carrier and chloropropyltriethoxysilane as a crosslinking agent to make a hydrophobic nanocellulose hydrogel with a low density (≤0.086 g·cm^−3^) and high porosity (≥95.2%).

Carbon nanotubes (CNTs) are widely regarded as one-dimensional nanomaterials because they have an inner diameter of about 1 nm, an outer diameter of a few to several tens of nanometers, a tube length in the micrometer range, and an aspect ratio of more than 100. Among them, multi-walled carbon nanotubes (MWCNTs) and MWCNT-based adsorbents have been explored for the removal of heavy metals from industrial effluents with high adsorption performance [18]. Jiang et al. [19] designed a double-layer aerogel structure with cellulose nanofibrils as the matrix and CNTs as the light-absorbing material, which could achieve a 76.3% solar energy conversion efficiency under the standard solar radiation. Zou et al. [20] prepared an ultralight free-standing MWCNT aerogel with an anisotropic macroporous honeycomb structure and a surface area of 580 m^2^·g^−1^.

In this work, cellulose/MWCNT-NH_2_ nanocomposite (NC-MWCNT-NH_2_) aerogels are prepared from cellulose nanofibers and MWCNTs-NH_2_ using a freeze-drying method for adsorption of oil and organic solvents. The different MWCNT contents are selected to evaluate the effect of the NC-MWCNT-NH_2_ structure on the adsorption property. The structure of as-prepared aerogels is analyzed using scanning electron microscope (SEM), Fourier transform infrared spectroscopy (FTIR), thermogravimetric analyzer (TGA), and specific surface and porosity analyzer (BET). The adsorption property of these aerogels for various oils and organics is measured in detail. It is found that the adsorption performance of NC-MWCNT-NH_2_ nanocomposite aerogels is increased first and then decreased with the increasing MMWCNT-NH_2_ content. The aerogel with 0.1 wt.% MWCNTs-NH_2_ shows the highest adsorption capacities for all the oils and organic solvents. The research demonstrates that the introduction of MWCNTs-NH_2_ can significantly improve the adsorption properties of aerogels and offers prospects for the application of cellulose aerogels in oil spill treatment.

## 2. Materials and Methods

### 2.1. Materials

Nanocellulose (CNF, diameter: 4–10 nm, length: 1–3 μm, 0.8 wt.% aqueous solution) was purchased from Guilin Qihong Technology Co., Ltd, Guilin, China. Aminated MWCNTs (outer diameter: 8–15 nm, length: ~50 μm) were provided by Jiangsu XFNANO Materials Tech Co., Ltd., Nanjing, China. Cyclohexane ethyl acetate, ethanol, and dichloromethane are all of analytical purity and were supplied by Shanghai Macklin Biochemical Technology Co., Ltd., Shanghai, China. Other chemicals were used as received without purification.

### 2.2. Preparation of Nanocellulose Aerogel

The preparation of pure NC aerogel was divided into three steps as a whole. First, a certain amount of aqueous nanocellulose solution was put in the de-ionized (DI) water to dilute the nanocellulose solution to 0.4 wt% of NC. Then, the above solution was poured into a high-speed mixer (4000 r·min^−1^) for 10 min. Finally, the solution was taken out into the centrifuge tube (7 mL). Then, the tube was put into the freezing dryer with the temperature set to −56 °C and freeze-dried for 24 h. The pure NC aerogel was marked as A0.

### 2.3. Preparation of NC-MWCNT-NH_2_ Nanocomposite Aerogels

The same procedure was applied to prepare NC-MWCNT-NH_2_ nanocomposite aerogels, in which the MWCNTs were firstly added into the NC solution and then put in the high-speed mixer for mixing. The aminated MWCNT content in the aqueous solution was 0.05, 0.1, and 0.15% in mass fraction, and the corresponding samples were named A1, A2, and A3, respectively. Contents of raw materials in nanocomposite aerogels are shown in Table 1.

### 2.4. Characterization of Aerogels

The surface morphology of aerogels was observed on SEM (Hitachi S-4800, Tokyo, Japan) with an acceleration voltage of 5 kV. The chemical structure of aerogels was conducted on the FTIR (IS20, Thermo Scientific, Thermo Nicolet NEXUS, Waltham, MA, USA) with an attenuated total reflection (ATR) accessory over the range from 4000 to 400 cm^−1^. Thermal degradation of aerogels was investigated by thermogravimetric analysis (TGA, Q50, TA Instruments, New Castle, DE, USA) with a heating rate of 10 °C min^−1^ in the air. The specific surface area of aerogels was determined using the Specific Surface Area Analyzer (Tristar3020, McMurray Tick Instruments Co., Ltd., Shanghai, China). The porosity (s) of aerogels was calculated using Equation (1) [21]:

(1) $$s=\frac{m_{2}-m_{1}}{m_{2}-m_{1}+m}$$

where *m_1_* is the mass of the sealed weighing vial filled with anhydrous ethanol after adsorption, *m_2_* is the mass of the sealed weighing vial filled with anhydrous ethanol before adsorption, and *m* is the mass of the aerogel used.

### 2.5. Adsorption Test

The adsorption tests were conducted as follows. Firstly, a piece of aerogel was weighed as *m_3_*. Secondly, the small weighing bottle with a lid containing 4 mL of ethyl acetate, anhydrous ethanol, dichloromethane, acetone, kerosene, pump oil, and used pump oil was weighed as *m_4_*. After that, the aerogel was put into oil for adsorption for 5 min without agitation. Then, the aerogel was quickly taken out and the mass of the bottle after adsorption was weighed as *m_5_*. The adsorption capacity (*A*) of oils could be computed as the weight difference between *m_5_* and *m_4_* divided by aerogel mass *m_3_* shown in Equation (2). The reported value is the average of three measurements with the error bars.

(2) $$A=\frac{m_{4}-m_{5}}{m_{3}}$$

## 3. Results

### 3.1. Structure Characterization

The morphology of MWCNTs-NH_2_ used in this work is characterized by SEM, and the results are shown in Figure S1. It presents a typical carbon nanotube morphological structure, showing a highly entangled tube structure with a diameter of about 10–20 nm. The as-prepared NC-MWCNT-NH_2_ aerogel with a density of about 6 mg/cm^3^, as shown in Figure S2, could be placed on the stamen of a flower without the stamen being deformed.

**Figure 1:**
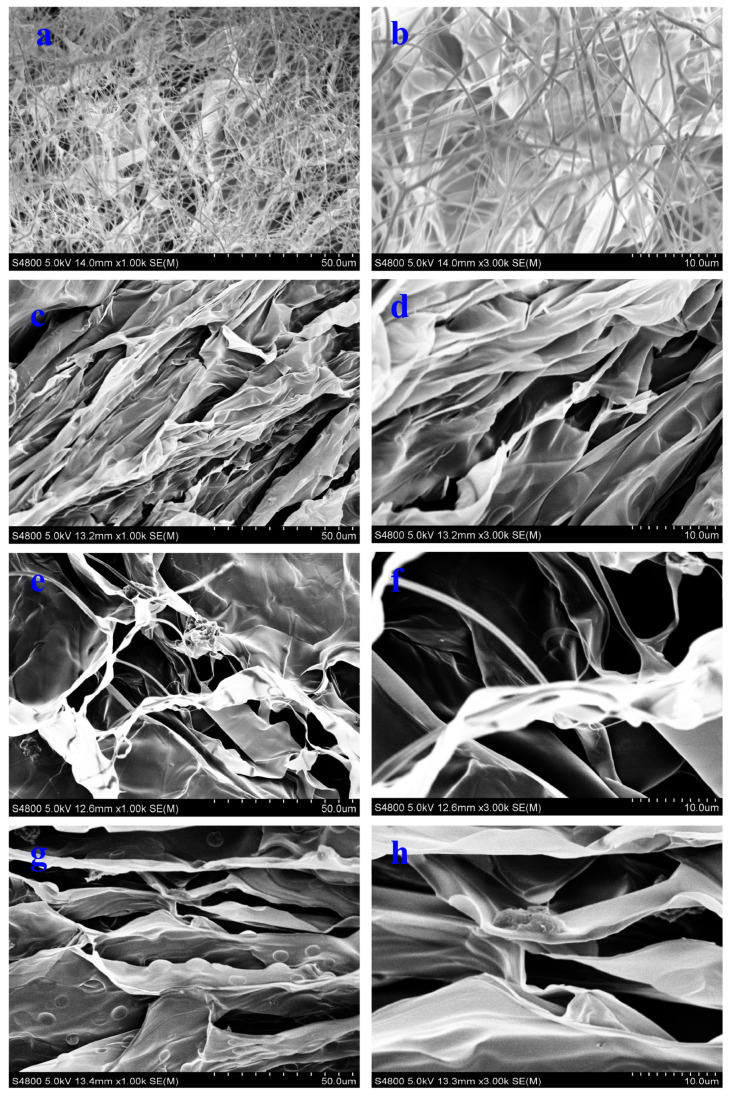
SEM images of (**a**,**b**) A0, (**c**,**d**) A1, (**e**,**f**) A2, and (**g**,**h**) A3.

The morphology of pure NC aerogel and NC-MWCNT-NH_2_ aerogel is carried out by SEM. In Figure 1a,b, the SEM images of the A0 sample show many fibrous thread-like structures with radial diameters of about 2–3 μm, which are coiled and entangled with each other, forming a three-dimensional network structure. There is a certain lamellar structure under the tread-like structure. The SEM images of the A1 sample reveal an obvious typical lamellar structure (Figure 1c,d). The structure of the A2 sample is relatively porous, and it can be observed that some filamentary fibers are still retained in its structure, and these filamentary fibers and MWCNTs-NH_2_ together build up the laminated structure of this composite aerogel (Figure 1e,f). The A3 sample has an overall layered structure (Figure 1g,h), which is also reported in the polyvinyl alcohol and CNF aerogel with a laminated structure [16,19].

**Figure 2:**
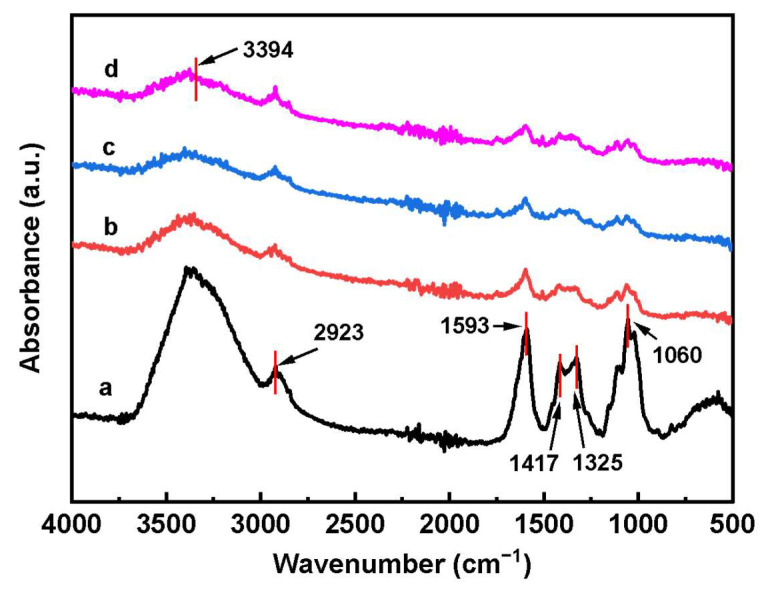
FTIR spectra of the aerogel samples: (a) A0, (b) A1, (c) A2, and (d) A3.

Figure 2 shows the FTIR spectra of A0, A1, A2, and A3. As illustrated in Figure 2, the absorption peaks at 1060, 1325, 1417, 1593, and 2923 cm^−1^ are assigned to the stretching vibration of C-O, -OH, CH_2_-OH, hydrogen bonding, and the saturated C-H bond in the cellulose structure, respectively [22]. These characteristic absorption peaks can also be observed in the NC-MWCNT-NH_2_ nanocomposite aerogels [23]. The FTIR spectrum of MWCNTs-NH_2_ is shown in Figure S3. The peak around 3394 cm^−1^ belongs to the -NH stretching vibration of MWCNTs-NH_2_ [24].

**Figure 3:**
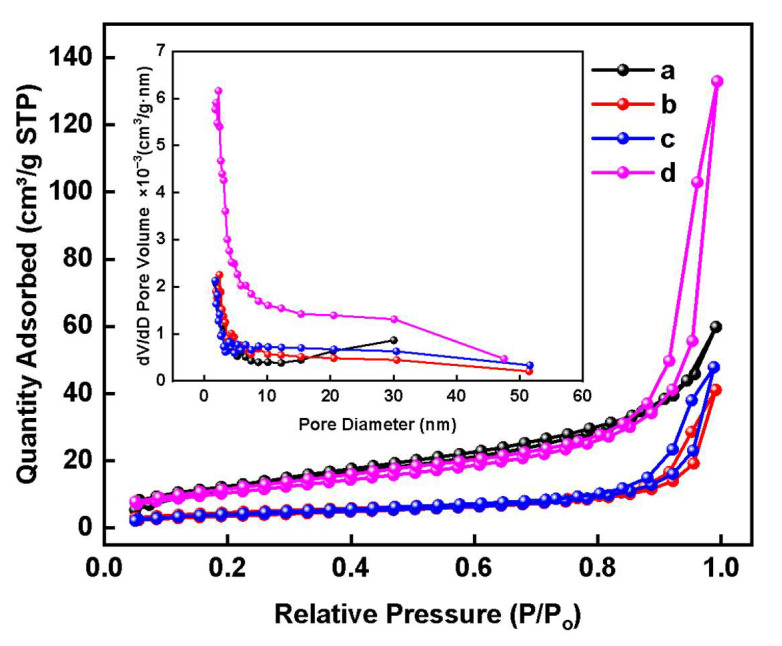
Nitrogen adsorption–desorption curves and pore size distribution curves (inset) of aerogel samples: (a) A0, (b) A1, (c) A2, and (d) A3.

BET analysis of pure NC aerogel and NC-MWCNT-NH_2_ nanocomposite aerogels with mass fractions of 0.05%, 0.1%, and 0.15%, respectively, was carried out. The nitrogen adsorption–desorption curves and pore size distribution curves of these aerogel samples are shown in Figure 3. In the inset of Figure 3, in the pore size distribution curves, the pore sizes of these samples are in the range of about 2–55 nm, but most of the pore sizes are distributed in the range of 2–15 nm. The nitrogen adsorption–desorption curves of these aerogels are in accordance with the type-IV adsorption isotherm, exhibiting mesoporous properties. As shown in Table S1, the resulting BET specific surface areas of A0, A1, A2, and A3 samples are 11.09, 13.42, 14.95, and 38.86 m^2^∙g^−1^, respectively. With the gradual increase in the mass fraction of MWCNTs-NH_2_ in the nanocomposite aerogel, its BET specific surface area is also gradually increased. The porosity of A0, A1, A2, and A3 samples is 96.27, 97.55, 97.80, and 96.81%, respectively, in which the NC-MWCNT-NH_2_ nanocomposite aerogel with an MWCNT-NH_2_ mass fraction of 0.1%, i.e., the A2 sample, has the largest porosity.

**Figure 4:**
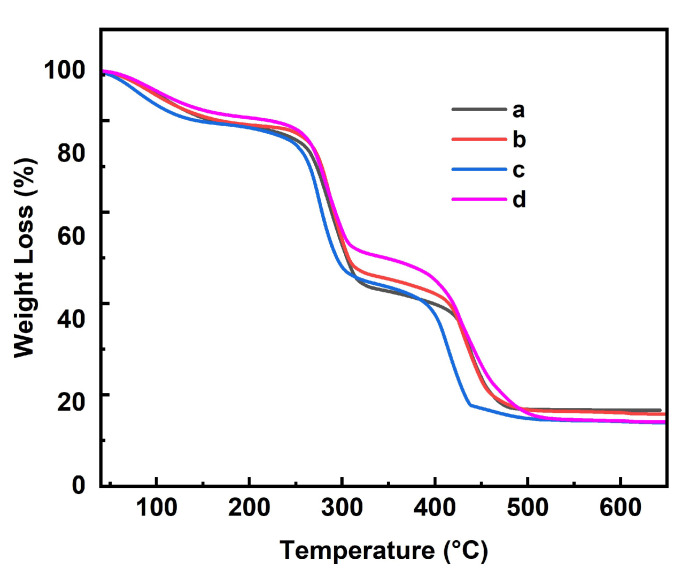
TGA curves of aerogel samples: (a) A0, (b) A1, (c) A2, and (d) A3.

To analyze the thermal stability of aerogels, TGA analysis was performed in the air condition in Figure 4. All four curves show about 10% weight loss before rising to 150 °C, arising from the evaporation of adsorbed water in the samples. The first stage of weight loss for the samples from 260 to 320 °C is due to the degradation of the 3D network structure of the nanofibrillar cellulose, and the variation in weight loss of pure NC aerogel at this stage is higher than that of the other three samples. The second stage of weight loss for the samples is in the range of 380 to 520 °C, which is caused by the thermal decomposition of the carbon skeleton of the aerogel. The variation in weight loss at this stage of the three nanocomposite aerogels is obviously higher than that of the pure NC aerogel for the addition of MWCNTs-NH_2_ as listed in Table S3. We also investigated the thermal stability of MWCNTs in air, and as seen in Figure S4, the MWCNTs-NH_2_ are thermally stable and begin to show rapid decomposition at temperature higher than 500 °C. Both A1 and A3 show a typical lamellar structure, so the TGA curves of samples are very similar, while the small amount of fiber filaments in sample A2 not only increases the porosity of sample, but also alters its weight loss.

### 3.2. Adsorption Performance

**Figure 5:**
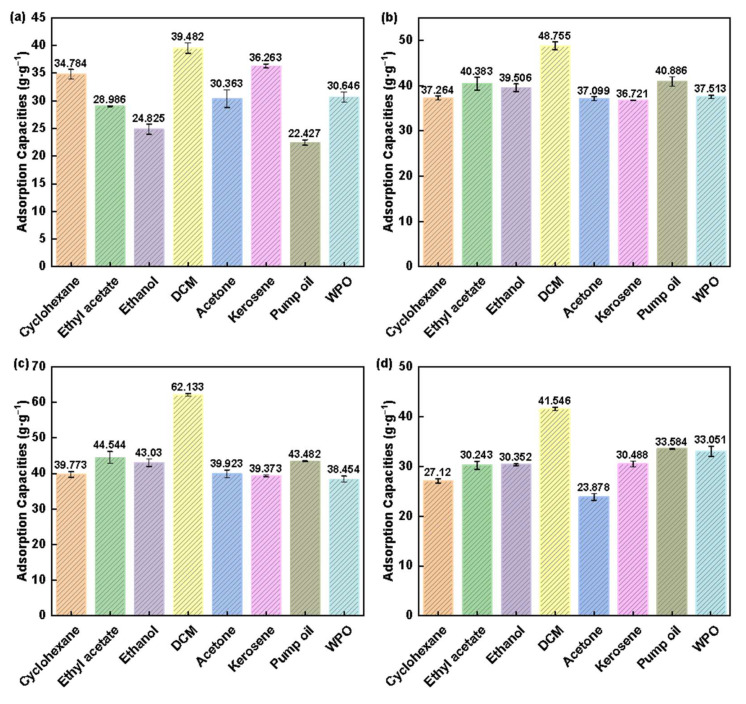
Adsorption of oils and organic solvents by different aerogels: (**a**) A0, (**b**) A1, (**c**) A2, and (**d**) A3, where DCM means dichloromethane, and WPO means wasted pump oil.

To investigate the adsorption properties of pure NC aerogel and NC-MWCNT-NH_2_ nanocomposite aerogels on oils and organic solvents, the cyclohexane, ethyl acetate, ethanol, dichloromethane, acetone, kerosene, pump oil, and wasted pump oil were selected for testing. As shown in Figure 5a, the adsorption capacity of the pure NC aerogel on cyclohexane, ethyl acetate, ethanol, dichloromethane, acetone, kerosene, pump oil, and wasted pump oil is 34.78 ± 0.87, 28.99 ± 0.08, 24.83 ± 0.92, 39.48 ± 0.9, 30.36 ± 1.59, 36.26 ± 0.36, 22.43 ± 0.48, and 30.65 ± 0.93 g·g^−1^, respectively. From the data in Figure 5b, NC-MWCNT-NH_2_ nanocomposite aerogels with 0.1% mass fraction of NC-MWCNT-NH_2_ have the best adsorption capacity, and its adsorption amounts of cyclohexane, ethyl acetate, ethanol, dichloromethane, acetone, kerosene, pump oil, and wasted pump oil are 39.77 ± 0.82, 44.54 ± 1.67, 43.03 ± 1.06, 62.13 ± 0.36, 39.92 ± 1.09, 39.37 ± 0.28, 43.48 ± 0.06, and 38.45 ± 0.84 g·g^−1^, respectively. The results are also listed in Table S4 for clarity. The adsorption capacity of pump oil is increased significantly, almost doubling. Overall, the adsorption performance of NC-MWCNT-NH_2_ nanocomposite aerogels was basically higher than that of the pure NC aerogel. It is noticed that the adsorption performance of NC-MWCNT-NH_2_ nanocomposite aerogels is increased first and then decreased with the increasing MWCNT-NH_2_ content. This may be the reason that, according to the adsorption data, sample A2 shows the highest adsorption capacities for all the oils and organic solvents. The results are consistent with the highest porosity of sample A2, and the laminated structure with filamentary fibers are remained in the nano composite aerogel. At the same time, all aerogels show a higher adsorption capacity for dichloromethane because the adsorption capacities of aerogels for organic solvents are affected by their density and surface tension. The greater the density and surface tension, the larger the adsorption capacities will be obtained [25].

As shown in Table 2, compared to other reported oil adsorbents, our NC-MWCNT-NH_2_ nanocomposite aerogels exhibit relatively higher adsorption capacities than those of sponges, foams, and inorganic aerogels [26,27,28,29], but lower than nanocellulose aerogels prepared by crosslinking individual cellulose nanofibers and the hydrophobic modification of the surface wettability [30].

## 4. Conclusions

In this work, the NC-MWCNT-NH_2_ nanocomposite aerogels with different contents of MWCNTs-NH_2_ are prepared, and the adsorption performance is studied in detail. The NC-MWCNT-NH_2_ nanocomposite aerogel with a MWCNT-NH_2_ mass fraction of 0.1% has the best adsorption performance with the adsorption capacity to cyclohexane, ethyl acetate, anhydrous ethanol, dichloromethane, acetone, kerosene, pump oil, and wasted pump oil of 39.77 ± 0.82, 44.54 ± 1.67, 43.03 ± 1.06, 62.13 ± 0.36, 39.92 ± 1.09, 39.37 ± 0.28, 43.48 ± 0.06, and 38.45 ± 0.84 g·g^−1^, respectively. These results are increased by 14.3, 53.7, 73.3, 57.4, 31.5, 8.6, 93.9, and 25.5%, respectively, compared with that of the pure NC aerogel. It can be found that the adsorption performance of the NC-MWCNT-NH_2_ nanocomposite aerogel increases and then decreases with the increase in MWCNT-NH_2_ content. The A2 sample exhibits the highest adsorption capacity for all oils and organic solvents. This result is consistent with the highest porosity and the retention of the lamellar structure of filamentous fibers in the nanocomposite aerogel.

In a word, the introduction of MWCNTs-NH_2_ into nanocellulose aerogels could significantly improve the structure of aerogels, which is more conducive to the adsorption of oils and organic matter.

## Figures and Tables

**Table 1:** Contents of raw materials in nanocomposite aerogels.

NO	MWCNT-NH_2_ Content (wt.%)	Cellulose Content (wt.%)	Mass Ratio of MWCNT-NH_2_ and Cellulose
A1	0.05	0.4	1:8
A2	0.1	0.4	2:8
A3	0.15	0.4	3:8
A0	0	0.4	0

**Table 2:** Comparison of properties for various oil-absorbing materials in the literature.

Materials	Oil Type	Adsorption Capacity (g·g^−1^)	Literature
BN/SiO_2_@PU	Chloroform	3	[26]
F-rGO@WS	Pump oil	3.63	[27]
Graphene aerogels	Diesel oil	25	[28]
Silica aerogels	Motor oil	15.1	[29]
Nanocellulose aerogels	Hexane	80	[30]
NC-MWCNT-NH_2_ aerogel	Pump oil	43.48	This work

## Data Availability

No data were used for the research described in the article.

## Supplementary Materials

The following supporting information can be downloaded at: https://www.mdpi.com/article/10.3390/polym17070869/s1, Figure S1: The morphology of MWCNTs-NH_2_; Figure S2: The resulting nanocomposite aerogel placed on the stamen of a flower; Figure S3: The FTIR spectrum of MWCNTs-NH_2_; Figure S4: The TGA curve of MWCNTs-NH_2_; Figure S5. SEM image of pure nanocellulose. Table S1: BET specific surface area and porosity of sample; Table S2: Adsorption of oils and organic solvents by different aerogels; Table S3. Variation of weight percentage of aerogels in TGA. (First stage, from 260 to 320 °C; Second stage, from 380 to 520 °C); Table S4 Adsorption of oils and organic solvents by different aerogels.
