# Supplementary material for: Cellulose/Aminated Multi-Walled Carbon Nanotube Nanocomposite Aerogels for Oil Adsorption
Source: Polymers (Basel). 2025 Mar 24;17(7):869. doi: 10.3390/polym17070869 (PMC11991409; doi:10.3390/polym17070869)
Supplement: Supplementary file 1 [file polymers-17-00869-s001.zip › polymers-3521943-supplementary.pdf]

Supplementary Information:

# Cellulose/Aminated Multi-Walled Carbon Nanotube Nanocomposite Aerogels for Oil Adsorption

**Runlin Han [1,†], Zihan Liu [2,†], Faxiang Feng [1], Shi Su [1], Guilin Dong [1], Xiaobing Liu [1] and Hongbo Gu [2,*]**

[1] Key Laboratory of Jiangxi Province for Special Optoelectronic Artificial Crystal Materials, School of Chemistry and Chemical Engineering, Jinggangshan University, Ji'an 343009, China; hanrunlin@163.com (R.H.); fengfaxiangfj@163.com (F.F.); 178885271280@163.com (S.S.); dong2990455445@163.com (G.D.); liuxiaobing805@163.com (X.L.)
[2] Key Laboratory of Chemical Assessment and Sustainability, School of Chemical Science and Engineering, Tongji University, Shanghai 200092, China; 15638112382@139.com
* Correspondence: hongbogu2014@tongji.edu.cn

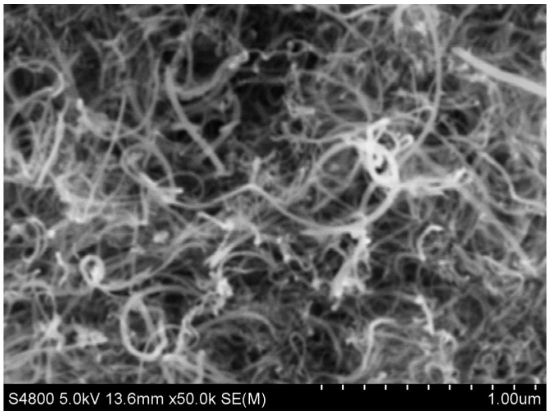


Figure S1 The morphology of aminated MWCNTs.

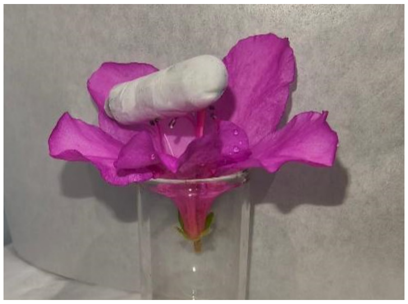

Figure S2 The resulting nanocomposite aerogel placed on the stamen of a flower.

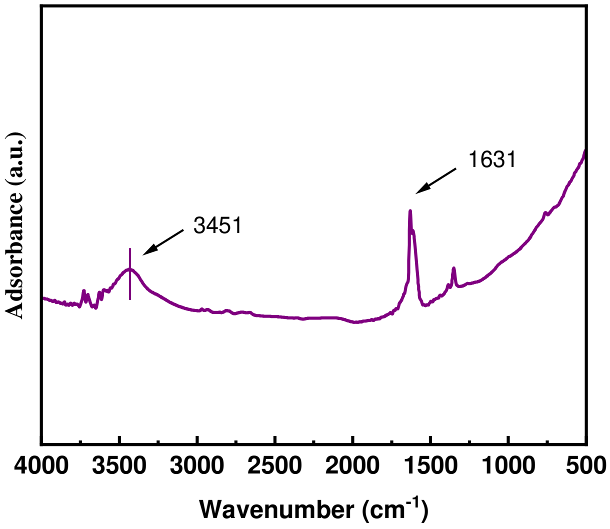


Figure S3 The FTIR spectrum of $MWCNTs-NH_2$.

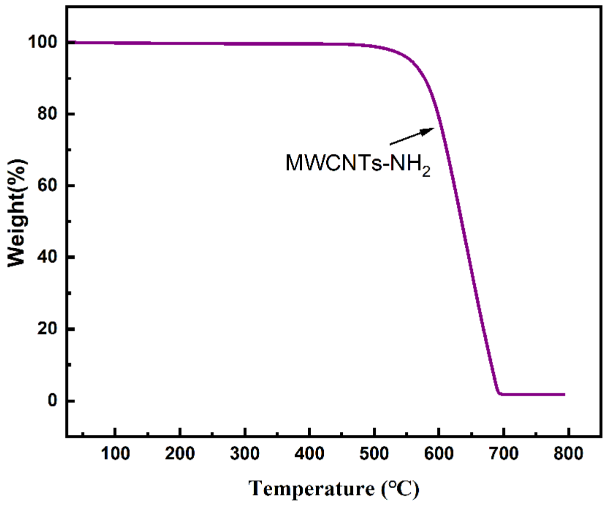


Figure S4 The TGA curve of $MWCNTs-NH_2$.

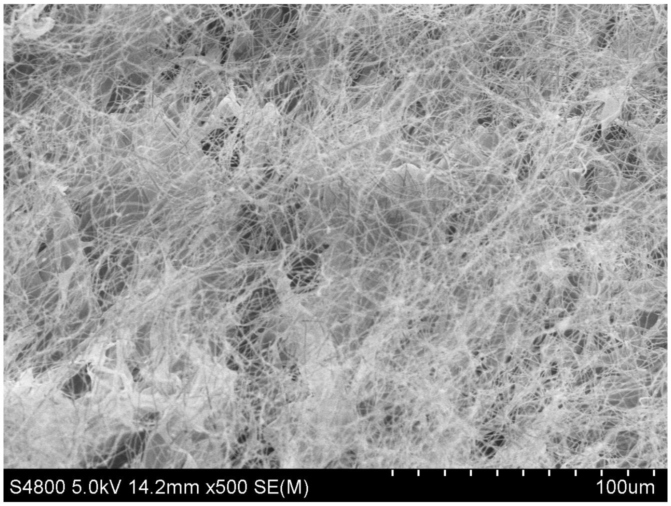


Figure S5 SEM image of pure nanocellulose.

Table S1. BET specific surface area and porosity of sample.

| Sample | BET ($m^2$ $g^{-1}$) | Porosity（%） |
| --- | --- | --- |
| A0 | 11.09 | 96.27 |
| A1 | 13.42 | 97.55 |
| A2 | 14.95 | 97.80 |
| A3 | 38.86 | 96.81 |

Table S2. The amount of aerogel samples used for adsorption.

| Oil type | A0 (g) | A1 (g) | A2 (g) | A3 (g) |
|---|---|---|---|---|
| cyclohexane | 0.0238 | 0.0223 | 0.0219 | 0.0289 |
| ethyl acetate | 0.0285 | 0.0248 | 0.0240 | 0.0286 |
| ethanol | 0.0240 | 0.0235 | 0.0247 | 0.0265 |
| dichloromethane | 0.0229 | 0.0330 | 0.0349 | 0.0362 |
| acetone | 0.0224 | 0.0229 | 0.0242 | 0.0324 |
| kerosene | 0.0248 | 0.0246 | 0.0254 | 0.0234 |
| pump oil | 0.0228 | 0.0265 | 0.0260 | 0.0249 |
| wasted pump oil | 0.0235 | 0.0279 | 0.0256 | 0.0278 |

Table S3. Variation of weight percentage of aerogels in TGA. (First stage, from 260 to 320 ℃; Second stage, from 380 to 520 ℃)

| Variation (%) | A0 | A1 | A2 | A3 |
|---|---|---|---|---|
| First stage | 35.2 | 33.1 | 32.8 | 29.6 |
| Second stage | 24.9 | 27.9 | 27.9 | 33 |

Table S4 Adsorption of oils and organic solvents by different aerogels.

| oil type | A0 ($g \cdot g^{-1}$) | A1 ($g \cdot g^{-1}$) | A2 ($g \cdot g^{-1}$) | A3 ($g \cdot g^{-1}$) |
|---|---|---|---|---|
| cyclohexane | 34.78±0.87 | 37.26±0.42 | 39.77±0.82 | 27.12±0.43 |
| ethyl acetate | 28.99±0.08 | 40.38±1.43 | 44.54±1.67 | 30.24±0.81 |
| ethanol | 24.83±0.92 | 39.51±0.86 | 43.03±1.06 | 30.35±0.17 |
| dichloromethane | 39.48±0.95 | 48.76±0.89 | 62.13±0.36 | 41.55±0.33 |
| acetone | 30.36±1.59 | 37.10±0.44 | 39.92±1.09 | 23.88±0.66 |
| kerosene | 36.26±0.36 | 36.72±0.03 | 39.37±0.28 | 30.49±0.57 |
| pump oil | 22.43±0.48 | 40.89±1.02 | 43.48±0.06 | 33.58±0.08 |
| wasted pump oil | 30.65±0.93 | 37.51±0.37 | 38.45±0.84 | 33.05±1.02 |
